# Boosting Synergistic Antioxidant and Anti-Inflammatory Properties Blending Cereal-Based Nutraceuticals Produced Using Sprouting and Hydrolysis Tools

**DOI:** 10.3390/foods13121868

**Published:** 2024-06-14

**Authors:** Iván Jesús Jiménez-Pulido, Ana Belén Martín-Diana, Irene Tomé-Sánchez, Daniel de Luis, Cristina Martínez-Villaluenga, Daniel Rico

**Affiliations:** 1 Agrarian Technological Institute of Castilla and Leon (ITACyL), Ctra. Burgos Km 119, Finca Zamadueñas, 47071 Valladolid, Spain; jimpuliv@itacyl.es (I.J.J.-P.); mardiaan@itacyl.es (A.B.M.-D.); 2 Department of Technological Processes and Biotechnology (DPTB), Institute of Food Science, Technology and Nutrition (ICTAN), Spanish National Research Council (CSIC), 28040 Madrid, Spain; i.tome@ictan.csic.es (I.T.-S.); c.m.villaluenga@csic.es (C.M.-V.); 3 Endocrinology and Clinical Nutrition Research Center (IENVA), Faculty of Medicine, University of Valladolid, Av. Ramón y Cajal, 3, 47003 Valladolid, Spain; dluisro@saludcastillayleon.es

**Keywords:** antioxidant, anti-inflammatory, oat, wheat, hydrolysis, sprouting, β-glucans, phytic acid

## Abstract

Nutraceuticals obtained from sprouted wheat and oat grains and processing by-products (bran and hull, respectively) naturally containing antioxidant and anti-inflammatory compounds were evaluated. The objective of this study was the development of a cereal-based nutraceutical formula combining extracts from sprouts and by-products and the exploration for potential synergetic effects in their bioactive properties. The antioxidant and anti-inflammatory capacities, glycemic index, phytic acid, and β-glucan of individual wheat bran hydrolysate (EH-WB), sprouted wheat (SW), oat hull hydrolysate (EH-OH), sprouted oat (SO), and combined ingredients (CI 1, CI 2, and CI3) were used to tailor an optimal nutraceutical formula. The three blend ingredients (CI 1, CI2, and CI3) were formulated at different ratios (EH-WB:SW:EH-OH:SO; 1:1:1:1, 2:1:2:1, and 1:2:1:2, *w*:*w*:*w*:*w*, respectively). The resulting mixtures showed total phenol (TPs) content ranging from 412.93 to 2556.66 µmol GAE 100 g^−1^ and antioxidant capacity values from 808.14 to 22,152.54 µmol TE 100 g^−1^ (ORAC) and 1914.05 to 7261.32 µmol TE 100 g^−1^ (ABTS^•+^), with Fe^3+^ reducing ability from 734. 02 to 8674.51 mmol reduced Fe 100 g^−1^ (FRAP) for the individual ingredients produced from EH-WB and EH-OH, where high antioxidant activity was observed. However, the anti-inflammatory results exhibited an interesting behavior, with a potentially synergistic effect of the individual ingredients. This effect was observed in CI2 and CI3, resulting in a higher ability to inhibit IL-6 and TNF-α than expected based on the anti-inflammatory values of their individual ingredients. Similar to the antioxidant properties, oat-based ingredients significantly contributed more to the anti-inflammatory properties of the overall mixture. This contribution is likely associated with the β-glucans and avenanthramides present in oats. To ensure the bioaccessibility of these ingredients, further studies including simulated digestion protocols would be necessary. The ingredient formulated with a 2:1 hydrolysate-to-sprout ratio was the most effective combination, reaching higher biological characteristics.

## 1. Introduction

Current lifestyles have a negative impact on daily habits, especially imbalanced diets which contribute to increased risk factors involved in the development of chronic diseases [1].

Over the past two decades, the amount of information available to society has significantly impacted on consumers’ awareness of the advantages in maintaining a correct and balanced diet [2,3]. A healthy diet is associated with substantial consumption of fresh and natural products, a high consumption of fiber and antioxidant compounds, and, additionally, a reduction in high-calorie foods. These low-nutrient foods increase oxidative stress and inflammation which have been linked to the development of chronic pathologies such as obesity and type 2 diabetes [4,5].

In this sense, refined flour consumption and bakery products have been highly questioned due to their negative impact on health. Different studies have reported how refined flour consumption is associated with high-glycemic-index products, which can lead to rapid spikes in blood sugar levels [6,7], insulin resistance, diabetes, and being overweight. Additionally, refined flours are stripped of essential nutrients and fiber during processing, leading to less-nutritious end products. On the other hand, many studies have demonstrated that the inclusion of whole-grain cereals in the diet is associated with a reduction in risk factors and, consequently, the prevention of pathologies such as metabolic syndrome, obesity, type 2 diabetes, and cardiovascular diseases [8]. Aune et al. [9] developed a meta-analysis based on 45 studies (64 publications) and observed that whole grain intake had a positive association with a reduction in the risk of coronary heart disease, cancer, and diabetes. These findings support dietary guidelines that recommend increased intake of whole grains to reduce the risk of chronic diseases and premature mortality. This preventive effect is mainly related to dietary fiber, phenolic compounds associated with dietary fiber, and other phytochemicals, which are present in whole grains [10]. For this reason, nowadays, consumers considering health concerns frequently choose whole grains as a food group that can provide health benefits and reduce certain health risks, probably associated with the relationship between fiber and health [11]. Although it is important for consumers to express skepticism towards any whole grain claims made on a product that *does not* use the Whole Grain Stamp, it is likely that more information needs to be divulged to society so that they can recognize the benefits of these products and be able to identify them on the label [12,13].

Wheat (*Triticum aestivum* L.) is one of the most widely consumed crops worldwide [14]. Wheat is extensively used in the food industry, with its main application being the production of refined flours. Wheat bran (WB), a by-product obtained during the production of refined flours, accounts for 15% of the grain [15]. The composition of the whole wheat grain (WG) and bran (WB) varies, containing between 10 and 20% of protein, 1.5 and 5% of fat, and 70 and 90% of carbohydrates, of which around 15–50% is dietary fiber [16].

Oat (*Avena sativa*) is listed among the 10 most produced cereal crops in the world, according to FAOSTAT data [17]. Oat consumption is mainly in the form of flour, cereal, or milk substitute [18], producing oat hull (OH) as a by-product, which represents 25–35% of the grain [19]. The composition of oat grain (OG) and oat hull (OH) varies between 3 and 12% of protein, 0.5 and 7% of fat, and 80 and 90% of carbohydrates, highlighting its content of dietary fiber (12–90%) and β-glucan (0.1–5%) [20]. By-products of both cereals, wheat and oat, are rich in fiber and present a high antioxidant and anti-inflammatory capacity due to the presence of compounds associated with fiber, such as phenolic compounds and β-glucan (in the case of oat). Fiber is able to regulate intestinal function, which promotes weight control and reduces the risk of chronic diseases [21]. In addition, soluble dietary fiber has the capacity to modulate blood glucose levels, reducing its absorption [22]. β-glucan contributes to reduced blood glucose and cholesterol levels. These benefits are attributed to the increased viscosity of the chyme, which reduces enzyme–nutrient interaction, and their interaction with bile acids, which are not reabsorbed in the ileum [23,24].

Other bioactive compounds associated with dietary fiber in cereals are phenolic compounds, which are bound to the structure of cellulose, hemicellulose, and lignin, thus reducing the bioaccessibility of these compounds [25]. Among the phenolic compounds associated with fiber, gallic acid, hydroxybenzoic acid, ferulic acid, *p*-coumaric acid, vanillic acid, and caffeic acid are the most abundant in cereals [26,27].

The bioaccessibility of these compounds is frequently limited, and their extractability is ineffective using traditional methods. For this reason, the application of biotechnological strategies such as sprouting or enzymatic hydrolysis allows the release of these compounds, improving their bioactivity. Sprouting is one of these strategies, which increases the biochemical and nutritional composition of the grains naturally [28,29,30]. In this sense, germination induces changes in the structure of the grains, increasing their digestibility, reducing anti-nutrients, improving the availability of carbohydrates, and thus increasing bioactivity [31,32]. Alternatively, enzymatic hydrolysis is another strategy, which, through the combination of cellulolytic and xylanolytic enzymes, can depolymerize the β-D-(1→4)-glucosidic and β-D-(1→4)-xylosidic bonds [33]. Different authors have determined that enzymatic hydrolysis allows the degradation of cell wall fibers, increasing the availability of phenolic compounds and β-glucan [20,25,34].

Nutraceuticals present different advantages compared to functional foods due to different reasons. One is associated with the fact that nutraceuticals are usually in a concentrated format, making it easier to obtain therapeutic doses of beneficial compounds compared to consuming large amounts of whole foods [35] and nutraceuticals can be tailored to target specific health conditions or goals, whereas functional foods may not always contain the necessary therapeutic ingredients in effective doses [36]. These properties allow the standardization of specific bioactive compounds, ensuring consistency in potency and efficacy [37,38].

Nutraceuticals often rely on rigorous scientific research and clinical trials to support their health claims, providing a higher level of evidence compared to some functional foods that rely on anecdotal evidence or traditional use [39], and offer a convenient and practical way to supplement the diet with specific nutrients or bioactive compounds that may be lacking or difficult to obtain from food sources [40]. For this reason, nutraceuticals offer a targeted and reliable way to support health and well-being, with a level of standardization and scientific validation that may be lacking in some functional foods.

The use of sprouts and hydrolyzed bran/hull present interesting characteristics from a nutraceutical and biological point of view for the development of nutraceuticals [41]. Most nutraceuticals are produced from a single raw material; however, the combination of different nutraceuticals can allow the formulation of nutraceutical formulas with synergistic activities that can work together to produce greater effects than when used individually. Some plant compounds can enhance the bioavailability of others, leading to improved absorption and utilization of nutrients and bioactive compounds. Combining plant-based nutraceuticals strategically can optimize bioavailability and efficacy [42].

This study has focused on the development of combined nutraceuticals obtained from the use of biotechnological strategies previously optimized by the authors [20,41,43], such as germination of whole grains and enzymatic hydrolysis or their by-products (wheat bran and oat hulls). The final aim was to amplify the synergistic benefits of individual ingredients and formulate a nutraceutical blend with improved health antioxidant and anti-inflammatory outcomes.

## 2. Materials and Methods

### 2.1. Chemicals

Folin–Ciocalteu reagent, gallic acid (GA), iron (III) chloride hexahydrate (FeCl_3_∙6H_2_O), 2,4,6-tripyridyl-triazine (TPTZ), iron (II) sulfate heptahydrate (FeSO_4_∙7H_2_O), 6-hydroxy-2,5,7,8-tetramethyl-2-carboxylic acid (Trolox), 2,20-diazobis-(2-aminodinopropane)-dihydrochloride (AAPH), fluorescein, 2,20-azinobis 3-ethylbenzothiazoline-6-sulfonic acid (ABTS^•+^), 2,2-diphenyl-1-picrylhydrazyl (DPPH), ascorbic acid, ammonium molybdate, trichloroacetic acid, sodium hydroxide, and sodium phosphate (NaH_2_PO_4_∙H_2_O) were obtained from Sigma-Aldrich, Co. (St. Louis, MO, USA). Glucose oxidase-peroxidase (GOPOD) and amyloglucosidase (EC 3.2.1.3) were provided by Megazyme (Wicklow, Ireland). Sodium acetate, glacial acetic acid, sulfuric acid, and hydrochloric acid were obtained from PanReac AppliChem (ITW Reagents, Darmstadt, Germany). Food grade enzyme UltraFlo XL and Viscoferm were kindly provided by Novozymes (Bagsværd, Copenhagen, Denmark).

### 2.2. Materials

Wheat grain (WG) and bran (WB) were kindly supplied by Emilio Esteban, S.A. (Valladolid, Spain). Wheat (*Triticum aestivum* L.) was harvested in 2020 in Valladolid. A dry milling process was applied to remove the bran from the endosperm. Both oat grain (OG) and hull (OH) were kindly provided by Sdad. Coop. Regional Ltda. Ribera del Duero (Burgos, Spain). Oat (*Avena sativa* L., var. Chimene) were harvested during the 2019–2020 season in Burgos. Chimene is a winter variety, with high productivity, and is characterized by a white grain and a high protein content. Oat hulls were mechanically separated under dry conditions from the grain. Samples were milled and stored in vacuum plastic bags until further analysis.

### 2.3. Samples Preparation and Extraction

Wheat grain (WG) and oat grain (OG) were germinated following the method described previously [20]. Grains were hygienized for 30 min by soaking in water containing 0.5% food grade sodium hypochlorite (*v*/*v*) at a ratio of 1:6 (*w*/*v*). The grains were rinsed with tap water to neutralize pH and soaked in distilled water for 4 h. Then, the grains were placed on trays and transferred to a germination chamber (Snijders Scientific, Tilburg, The Netherlands) for 5 days at 21 °C with a relative humidity > 90%. Subsequently, the wheat and oat sprouts obtained (SW and SO, respectively) were treated with a high-pressure process (HPP) (Wave 600/135, Hiperbaric, Burgos, Spain) for 5 min at 600 MPa and freeze-dried (LyoBeta, Telstar, Barcelona, Spain).

Wheat bran (WB) and oat hull (OH) were subjected to an enzymatic hydrolysis treatment following a previously described method [20], with slight modifications. WB and OH were resuspended in water at a ratio of 1:20 (*w*/*v*), pH was adjusted to 5 with malic acid, and 1% enzyme (UltraFlo XL for WB and Viscoferm for OH) was added by dry weight of WB or OH (*w*/*w*). The mixture was then incubated in a bath at 47 °C for 20 h with agitation (Unitronic Vaivén C, Selecta S. A., Abrera, Spain). After incubation, the enzymes were inactivated in a bath at 95 °C for 5 min and subsequently treated with HPP at 600 MPa for 5 min. The insoluble fraction of the resulting hydrolysates, EH-WB and EH-OH, was separated using a nylon filter (200 µm mesh) and the hydrolysate was freeze-dried (LyoBeta, Telstar, Barcelona, Spain). 

**Scheme 1:**
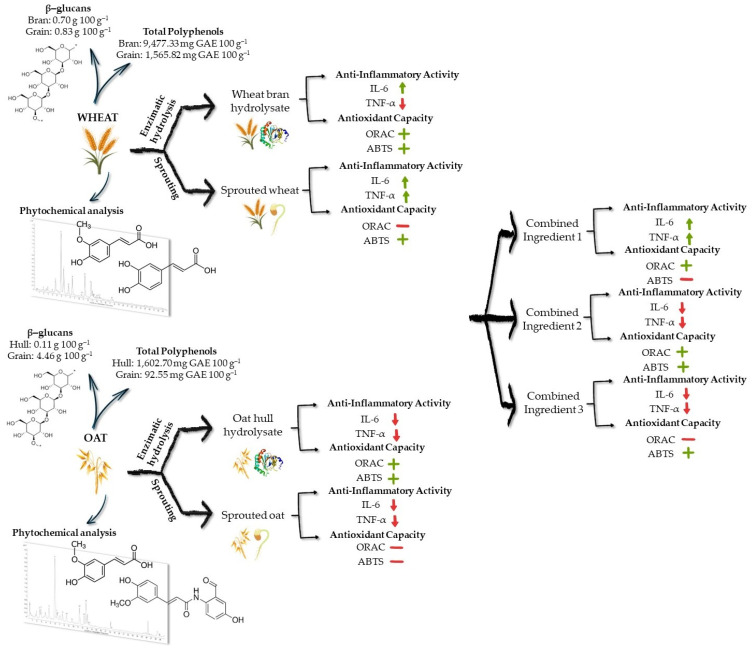
Scheme of antioxidant and anti-inflammatory activity of individual and combined ingredients. The ingredients wheat bran hydrolysate (EH-WB), sprouted wheat (SW), oat hull hydrolysate (EH-OH), and sprouted oat (SO) were all milled to a particle size of 0.5 mm. Thereafter, various combinations of ingredients were prepared with different proportions by weight of each ingredient, as described in Table 1.

Scheme 1 shows a diagram of antioxidant and anti-inflammatory activity of the individual and combined ingredients.

For extraction, one gram of sample was weighed, 10 mL of methanol/distilled water (50% *v*/*v*) at pH 2 was added and incubated at room temperature for 30 min. Samples were centrifuged at 4000 rpm for 10 min at 4 °C and the supernatant was filtered. The pellet was resuspended in 8 mL of methanol/distilled water (50% *v*/*v*) at pH 2 and incubated again for 30 min. The samples were centrifuged a second time, and the supernatant was filtered over the previous supernatant. Each sample was made up to a final volume of 20 mL with methanol/distilled water (50% *v*/*v*) at pH 2 and then aliquoted. Samples were stored at −80 °C until further analysis.

### 2.4. Total Phenol (TPs) Content

Total phenol (TPs) content was determined according to the method described by Slinkard and Singleton [44], using Folin–Ciocalteu phenol reagent. A standard curve was prepared with gallic acid (700–98 μM) for the quantification of the TP content of the samples. A microplate reader (Fluostar Omega, BMG, Ortenberg, Germany) was used to measure the absorbance of the standards and samples at 765 nm. Results were indicated as μmol gallic acid equivalents (GAE) 100 g^−1^ d.m. All analyses were performed in duplicate.

### 2.5. Total Antioxidant Capacity (TAC)

Total antioxidant capacity (TAC) was determined, for duplicates, with the oxygen radical absorbance capacity (ORAC), ABTS^•+^ radical cation scavenging activity, DPPH radical scavenging activity, and ferric reducing antioxidant power (FRAP) methods.

#### 2.5.1. Oxygen Radical Absorbance Capacity (ORAC)

ORAC assay was performed according to the method previously described by Ou et al. [45], with some modifications. A standard Trolox curve (7.5–210 μM) and samples were diluted with phosphate buffer (75 mM, pH 7.4). Twenty-five μL of sample, Trolox standard, and phosphate buffer as a blank were added to a black 96-well microplate with 125 μL of fluorescein and incubated at 37 °C for 3 min. Afterwards, 25 μL of AAPH solution was added to start the oxidation reaction and fluorescence was monitored for 150 min using a microplate reader (CLARIOstar Plus, BMG, Ortenberg, Germany) with a 485 nm excitation and 520 nm emission filters. To calculate the results, the areas under the fluorescein decay curves were plotted as a function of Trolox concentration. Results were reported as μmol TE 100 g^−1^ sample of dry matter (d.m.).

#### 2.5.2. ABTS^•+^ Radical Cation Scavenging Activity

The ABTS^•+^ assay was carried out following the method described by Re et al. [46], adapted by Martin-Diana et al. [47]. In a 96-well microplate, a volume of 20 μL of sample or standards was added and mixed with 200 μL of ABTS^•+^ working solution. The absorbance was determined at 734 nm after 60 min incubation with a microplate reader (Spectrostar Omega, BMG Ortenberg, Germany). As standard, a Trolox curve (7.5–210 μM) was used. Results were expressed as μmol TE 100 g^−1^ sample of d.m.

#### 2.5.3. DPPH Radical Scavenging Activity (DPPH)

The DPPH assay was performed based on Brand-Williams et al. [48], with slight modifications. The sample was mixed with miliQ water and DPPH working solution (120 μM in pure methanol) at a 1:4:5 volume ratio in a 96-well microplate. The absorbance was measured at 515 nm after 30 min incubation with a microplate reader (Spectrostar Omega, BMG, Ortenberg, Germany). A Trolox curve was used as standard (7.5–210 μM). Results were expressed as μmol Trolox equivalents (TE) 100 g^−1^ sample of d.m.

#### 2.5.4. Ferric Reducing Antioxidant Power (FRAP)

FRAP was evaluated according to the method described by Benzie and Strain [49], with slight modifications [16]. For the preparation of the FRAP working solution, acetate buffer (300 mM, pH 3.6), TPTZ solution (10 mM in 40 mM HCl), and FeCl_3_∙6H_2_O solution (20 mM) were mixed in a 10:1:1 volume ratio. As standard, a curve of FeSO_4_∙7H_2_O (400–3000 μM) was used. Then, 20 μL of sample and standard or distilled water as a blank were mixed in Eppendorf tubes with 1.9 mL of FRAP working solution. Tubes were stirred and incubated for 5 min. Absorbance was detected at 593 nm in a 96-well plate using a microplate reader (Spectrostar Omega, BMG Ortenberg, Germany). Results were expressed as mmol Fe^2+^ equivalents (FeE) 100 g^−1^ sample of d.m.

#### 2.5.5. Relative Antioxidant Capacity Index (RACI)

The relative antioxidant capacity (RACI) of the samples was used as an integral concept which allows the comparison of antioxidant capacity derived from different chemical methods [50]. RACI values were determined through the following equation: (x − µ)/σ, where x is the antioxidant value, µ is the average value of the results of the corresponding method (DPPH, ORAC, ABTS, and FRAP), and σ is the standard deviation.

### 2.6. Total Starch Content (TSC) and Estimated Glycemic Index (GI)

Total starch content (TSC) was determined using a K-TSTA-100A assay kit (Megazyme, Wicklow, Ireland). In tubes, 0.2 mL of 80% ethanol and 2 mL of dimethylsulfoxide (DMSO) were added to 100 mg of sample. Tubes were shaken and placed in a bath at 95 °C for 5 min. Subsequently, 3 mL of α-amylase diluted in a MOPS buffer (50 mM, pH 7) was added and placed in a bath at 100 °C for 12 min, being shaken every 2 min. Then, 4 mL of sodium acetate buffer (200 mM, pH 4.5) with 5 mM CaCl_2_ and 0.1 mL of amyloglucosidase was added. The tubes were placed in a bath at 50 °C for 30 min. After this time, the tubes were tempered, and the volume was adjusted to 100 mL with sodium acetate buffer. A 2 mL aliquot was centrifuged at 13,000 rpm for 5 min. A total of 20 µL of the supernatant was collected, mixed with 600 µL of GOPOD, and incubated at 50 °C for 20 min. The absorbance was measured at 510 nm using a 96-well plate in a microplate reader (Spectrostar Omega, BMG Ortenberg, Germany). Results were expressed as g starch 100 g^−1^ sample of d.m.

The determination of the estimated glycemic index (GI) was performed as described by Gularte and Rosell [51], with some modifications. Samples were dissolved with 50 mg of available starch in 2 mL of Tris-maleate buffer (0.1 M, pH 6) and then 2 mL of enzyme solution, containing porcine pancreatic amylase (460 U mL^−1^) and amyloglucosidase (6.6 U mL^−1^), was added. Aliquots of 150 µL were taken at 0, 10, 20, 30, 60, 90, and 120 min and the enzyme reaction was immediately stopped for 5 min in boiling water and cooled on ice. Subsequently, a volume of 150 µL of absolute ethanol was added and the sample was centrifuged (10,000× *g*, 5 min). The precipitate was washed with 200 µL EtOH:H_2_O (1:1, *v*/*v*) and the supernatants were mixed. A GOPOD kit (Megazyme, Bray, Ireland) was used for colorimetric analysis of glucose. Hydrolysis index (HI) and glycemic index (GI) values were determined using the formulas suggested by Grunfeld [52].

### 2.7. Determination of β-Glucan and Phytic Acid (PA)

β-glucan content was quantified by a mixed-linkage [(1-3)(1-4)]-β-glucan kit (Megazyme, Ireland), following the manufacturer’s instructions. The assay hydrolyses β-glucan to glucose using lichenase and β-glucosidase. For this purpose, approximately 100 mg of sample was weighed into a tube and 0.2 mL of 50% ethanol/distilled water (*v*/*v*) and 4 mL of sodium phosphate buffer (20 mM, pH 6.5) were added. The tube was shaken and incubated at 100 °C for 1 min, shaken again, and incubated at 100 °C for a further 2 min. The tube was stirred again with a vortex and incubated at 50 °C for 5 min. After this time, 0.2 mL of lichenase was added, stirred, and incubated at 50 °C for 1 h, stirring 3–4 times during this time. Subsequently, 5 mL of sodium acetate buffer (200 mM, pH 4) was added and stirred. It was tempered and centrifuged at 1000× *g* for 10 min. Then, 0.1 mL of the sample supernatant was added to three Eppendorf tubes, and 0.1 mL of β-glucosidase was added to two of them and 0.1 mL of sodium acetate buffer (50 mM, pH 4) to the other one (sample blank). The tubes were incubated at 50 °C for 10 min. A reaction blank and a standard were prepared with 0.1 mL of distilled water and 0.1 mL of the D-glucose standard from the kit, respectively. To both, 0.1 mL of sodium acetate buffer (200 mM, pH 4) was added. Finally, 3 mL of GOPOD was added to each tube, stirred, and incubated at 50 °C for 20 min. The absorbance was measured at 510 nm in a microplate reader (Spectrostar Omega, BMG Ortenberg, Germany). All measurements were performed in duplicate. Results were expressed as g β-glucan 100 g^−1^ d.m.

Phytic acid (PA) was determined using a K-PHYT assay kit (Megazyme, Wicklow, Ireland). Briefly, 0.5 g of sample was weighed into a tube, followed by the addition of 10 mL of 0.66 M HCl. The mixture was then left to shake overnight. The next day, 1 mL was taken from the tube and centrifuged at 13,000 rpm for 10 min. Then, 0.5 mL of the supernatant was taken and 0.5 mL of 0.75 M NaOH was added. Two tubes were prepared for each sample, one for free phosphorus and the other for total phosphorus analysis. In the tube designated for free phosphorus analysis, 0.62 mL of distilled water, 0.2 mL of solution 1 from the kit, and 50 µL of sample were added. In the tube for total phosphorus analysis, 0.6 mL of distilled water, 0.2 mL of solution 1 from the kit, 50 µL of sample, and 20 µL of phytase (kit) were added. The tubes were vortexed and incubated at 40 °C for 10 min. Subsequently, 20 µL of distilled water and 20 µL of kit solution 3 were added to the free phosphorus tube; and 20 µL of kit solution 3 and 20 µL of alkaline phosphatase (kit) were added to the total phosphorus tube. The tubes were vortexed and incubated at 40 °C for 15 min. After this time, 0.3 mL of 50% trichloroacetic acid was added and centrifuged at 13,000 rpm for 10 min. The coloring reagent was prepared by mixing in a 5:1 ratio of ascorbic acid (10% *m*/*v* with 1 M sulfuric acid) and ammonium molybdate (5% *m*/*v*), respectively, and a phosphorus standard curve (0–7.5 µg). In another tube, 1 mL of sample/standard was mixed with 0.5 mL of coloring reagent, and the tubes were incubated at 40 °C for 1 h. Subsequently, absorbance was measured at 655 nm with a microplate reader (Spectrostar Omega, BMG Ortenberg, Germany). Results were expressed as g phytic acid 100 g^−1^ of d.m. All measurements were performed in duplicate.

### 2.8. Determination of Anti-Inflammatory Activity

The RAW264.7 murine macrophage cell line was purchased from the American Type Culture Collection (Manassas, VA, USA) and cultured in complete DMEM (Dulbecco’s Modified Eagle Medium) supplemented with 10% (*v*/*v*) heat-inactivated fetal bovine serum and 1% penicillin/streptomycin (Life Technologies, Carlsbad, CA, USA), and maintained at 37 °C with 5% CO_2_. Cells in the logarithmic growth phase were seeded at a density of 5 × 10^4^ cells per well in 96-well plates containing complete DMEM and allowed to attach overnight. Subsequently, the complete DMEM was replaced with growth medium containing sterile-filtered samples diluted at a concentration of 0.5 mg/mL, along with 0.1 µg/mL of lipopolysaccharide from *Escherichia coli* O55:B5 (Sigma-Aldrich, St. Louis, MO, USA) and incubated for 24 h.

After the incubation period, the spent medium from the treated RAW264.7 cells was collected and stored at −80 °C for cytokine quantification. Then, cell viability was assessed using the CellTiter 96^®^ AQueous One Solution Cell Proliferation Assay (Promega, Madison, WI, USA).

The quantification of IL-6, TNF-α, MCP-1, and MIP-2 mouse cytokines from the collected cell culture medium was conducted simultaneously using the Mouse Cytokine Magnetic kit (Milliplex MCYTOMAG-70K-05, Merck Life Sciences, Madrid, Spain) according to the manufacturer’s instructions. Data were collected using a Luminex XYP flow cytometer (Luminex Co., Austin, TX, USA) and analyzed with BelysaTM Data Analysis Software (version 1.2). MKC cytokine determination was excluded from the analysis as its values were below the lower threshold in all analyzed samples.

### 2.9. Statistical Analysis 

Data were expressed as mean ± standard deviation. Analysis of variance (ANOVA) and post hoc Duncan’s test (*p* ≤ 0.05) were performed to identify differences between mean values. Statgraphics Centurion XVI^®^ software (StatPoint Technologies, Inc., Warrenton, VA, USA) was used to perform the statistical analyses. To elucidate the relationships between variables, a principal component analysis (PCA) was performed.

## 3. Results and Discussion

### 3.1. Total Phenol (TPs) Content

**Figure 1:**
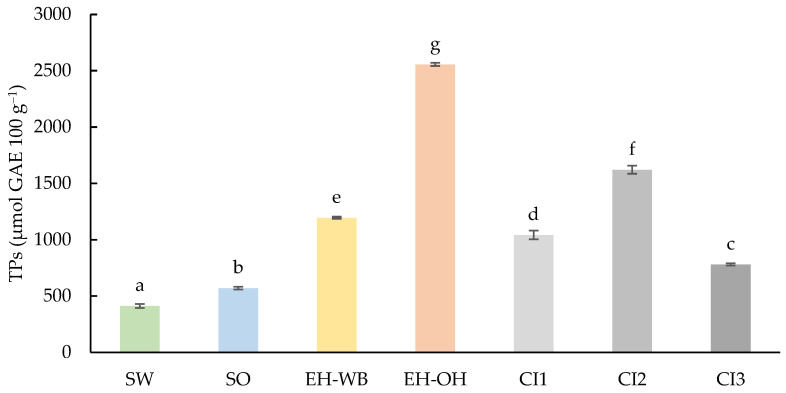
Total phenol (TPs) content of individual (SW, SO, EH-WB, and EH-OH) and combined ingredients (CI1, CI2, and CI3). Results are expressed as µmol GAE 100 g^−1^ of d.m. Mean values are represented as bars, and standard deviations are represented as error bars. Different letters indicate significant differences from each other (one-way ANOVA, post hoc Duncan’s test, *p* ≤ 0.05). Abbreviations: sprouted wheat (SW), sprouted oat (SO), wheat bran hydrolysate (EH-WB), oat hull hydrolysate (EH-OH), combined ingredient 1 (CI1), combined ingredient 2 (CI2), and combined ingredient 3 (CI3).

The total phenol content was determined in all individual (SW, SO, EH-WB, and EH-OH) and combined ingredients (CI1, CI2, and CI3; Figure 1). Individual ingredients showed significant differences (*p* < 0.05) between them, ranging in values from 412.93 to 2556.66 µmol GAE 100 g^−1^. The ingredients produced using hydrolyses exhibited the highest TP content compared to those produced using sprouting, and oat ingredients had higher TP values than wheat ingredients, regardless of the technological treatment used (hydrolysis or sprouting) (Figure 1). The germination of oat grains increased the TP content 20% more than that of wheat grains and the application of oat hull after hydrolytic process had double the TP compared to bran wheat. The results agreed with studies reported previously by the authors where optimized sprouting and enzymatic hydrolysis technologies enhanced the bioaccessibility of phenolic compounds, associated with better extractability and solubilization during both processes, but specially after the hydrolytic enzymatic procedures [20,30,53].

The TP values of the ingredients obtained using hydrolysis (EH-WB and EH-OH) ranged from 1000 to 2000 µmol GAE 100 g^−1^ for hydrolyzed bran/hull and were similar to values reported in [20,41]. The observed increment of phenolic content after the use of enzymes is due to the hydrolysis of phenolic compounds attached to tannins, lignin, cellulose, and proteins, which are the main structural components of bran and aleurone [54].

On the other hand, the TP values obtained using sprouting were similar to values reported previously by the authors (600 µmol GAE 100 g^−1^ [20]), although they were lower than values published by other authors, where 3-fold higher values (1800 to 3000 µmol GAE 100 g^−1^) have been reported [55]. These differences in TP could be associated with different aspects, the most significant of which are the genotype and environmental aspects which can modulate the phenol content [56,57]. Also, the conditions used during germination affect the TP content due to the degradation of the cellular structures of the grains by the enzymes, which favors the extractability of the bound phenolic compounds, and the process improves the de novo biosynthesis in the embryo axis of the phenolic content [41,58,59,60]. Moreover, humidity plays an important role in the process before (pre-sowing) and during the germination, as hydration involves the production of reactive oxygen species (ROS), which could damage the structures of DNA, proteins, lipids, and other macromolecules in the seeds. However, ROS scavenging is also pivotal for seed germination under stress conditions and comprises non-enzymatic components, mainly linked with overproduction of antioxidants [61]. Since hydration was maintained by capillarity, probably some of the solubilized phenols were released from the seeds to the water tank, resulting in a reduction in TP in the seed [62].

When these individual ingredients were combined (Table 1) the TP content of the final ingredients showed TP values from 781.17 to 1621.093 µmol GAE 100 g^−1^. 

When the ingredients were combined at the same ratio (CI1), the TP content was proportional to their contribution without any increment or decrease associated with their interaction. The same behavior was observed in the case of the combined ingredient formulated using a double concentration of hydrolyzed components compared to sprouts (CI2). However, in the case of CI3, the combined ingredient formulated using a double concentration of 2:1 sprouts/hydrolyzed component, respectively, the TP values reduced 30% with respect to the expected values. Since the ingredients were formulated after the extraction, it is unprovable that the reason for the reduction could be the interaction between different macromolecules; probably, the reason is associated with the fact that CI3 contains a higher concentration of peroxidases than CI1 and CI2, since the sprouts were in a 2:1 ratio. Peroxidases are enzymes present at a high concentration during germination and play a crucial role in the oxidation of phenols. The reaction proceeds through the formation of a radical intermediate, which then undergoes further reactions to form quinones and other oxidized products. This process of oxidation not only changes the chemical structure of the phenol but also makes it more reactive and readily available for further reactions, reducing the bioavailability of phenols [63]. 

Also, germination involves the degradation of starch into simple carbohydrates, which can interact with polyphenols and potentially reduce their activity in in vitro assays. This interaction may involve the formation of complexes between the two compounds, which could impact the bioavailability and effectiveness of the polyphenols, and, additionally, carbohydrates can compete for binding sites with polyphenols, leading to decreased activity [64,65].

### 3.2. Total Antioxidant Capacity (TAC)

TAC determinations of the individual ingredients and the combined ingredients were performed to assess the activity against different radicals (AAPH, ABTS^•+^, DPPH), and the reducing power was evaluated using FRAP assay. It is important to use multiple methods to measure total antioxidant activity in order to obtain a more comprehensive and accurate assessment. Different methods may capture different aspects of antioxidant activity, and using a combination of assays can provide a more robust measurement [66].

**Figure 2:**
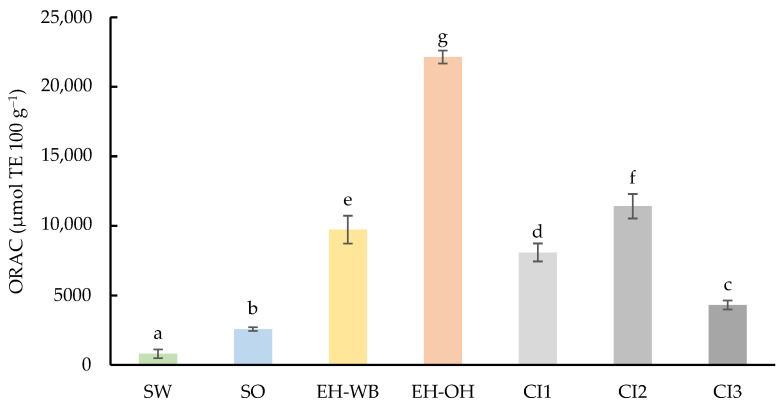
ORAC values of individual (SW, SO, EH-WB, and EH-OH) and combined ingredient (CI1, CI2, and CI3) samples. Results are expressed as µmol TE 100 g^−1^ of sample. Mean values are represented as bars, and standard deviations are represented as error bars. Different letters indicate significant differences from each other (one-way ANOVA, post hoc Duncan’s test, *p* ≤ 0.05). Abbreviations: sprouted wheat (SW), sprouted oat (SO), wheat bran hydrolysate (EH-WB), oat hull hydrolysate (EH-OH), combined ingredient 1 (CI1), combined ingredient 2 (CI2), and combined ingredient 3 (CI3).

The mechanism against the AAPH radical, involving the ability to scavenge peroxyl groups through the transfer of hydrogen atoms, was assessed using the ORAC method [67] in the individual and combined ingredients (Figure 2). The ORAC values obtained for the ingredients were higher than those reported in other studies that evaluated the effect of germination or enzymatic hydrolysis in relation to untreated grains or bran [20,68], probably related to the genotype, environmental aspects, and specific germinative and hydrolytic process.

The results obtained ranged from 808.14 to 22,152.54 µmol TE 100 g^−1^, showing significant (*p* < 0.05) differences among ingredients produced using sprouting or hydrolysis. EH-OH showed double the antioxidant capacity of the ingredient produced from wheat hydrolysis, with the ingredients obtained from sprouts showing values 10 times lower. The use of germination or hydrolysis for oat was more effective than wheat in terms of antioxidant activity, doubling the ORAC values when the ingredient was produced from germination and tripling it in the case of ingredients produced from byproduct hydrolysis.

The combined ingredients reduced the antioxidant capacity in comparison with the individual ingredients by between 20 and 57%. The highest values were observed in CI2 followed by CI1 and the ingredient with double the ratio of sprouts to hydrolyzed components had the lowest ORAC values were observed. The highest reduction in ORAC values was observed in CI3, at close to 50%.

When starch is hydrolyzed, it breaks down into smaller molecules such as sugars. Many of these smaller molecules are easily oxidized and can react with the reagents used in ORAC assays, thus causing a reduction in ORAC values [69].

**Figure 3:**
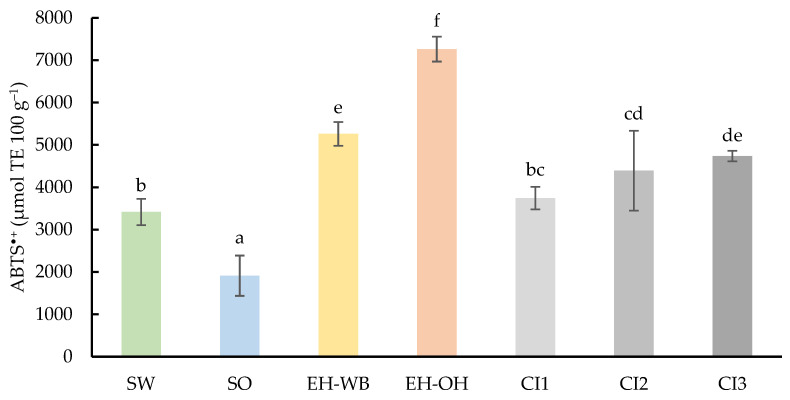
ABTS^•+^ values of individual (SW, SO, EH-WB, and EH-OH) and combined ingredient (CI1, CI2, and CI3) samples. Results are expressed as µmol TE 100 g^−1^ of sample. Mean values are represented as bars, and standard deviations are represented as error bars. Different letters indicate significant differences (*p* < 0.05). Abbreviations: sprouted wheat (SW), sprouted oat (SO), wheat bran hydrolysate (EH-WB), oat hull hydrolysate (EH-OH), combined ingredient 1 (CI1), combined ingredient 2 (CI2), and combined ingredient 3 (CI3).

Figure 3 shows the values obtained for the different ingredients and combinations of ingredients against the ABTS^•+^ radical. The ABTS^•+^ radical assay evaluates the ability of antioxidants to mediate electron transfer reactions [70]. Significant (*p* < 0.05) differences were observed among the different ingredients with values ranging from 1914.05 to 7261.32 µmol TE 100 g^−1^. The results from ABTS were higher in hydrolyzed ingredients compared to the sprout ingredients; however, the differences in antioxidant activity between them were lower than those observed when measured with ORAC, especially in wheat. These differences could be associated with the higher specificity of this assay for peptides, especially those with aromatic amino acids, such as tyrosine and tryptophan, which are often found to have strong antioxidant activity due to their ability to donate hydrogen atoms to free radicals. Peptides with sulfur-containing amino acids, such as cysteine and methionine, may also exhibit antioxidant activity by forming complexes with metal ions that can generate reactive oxygen species [71]. Probably, the ABTS assay takes into account the peptides produced from the increase in digestibility that occurs during the germination, which makes a significant contribution to the ABTS values. This effect was observed also in the case of the combined ingredients, where it was observed that the ingredient formulated with a ratio of 2:1 of sprout ingredients showed the highest activity (CI3), probably associated with its high content of hydrolyzed peptides. 

The combined ingredients showed values between 3744.06 and 4736.51 µmol TE 100 g^−1^, with a higher contribution from the hydrolyzed ingredients (EH-WB and EH-OH) in agreement with findings reported by Tomé-Sánchez et al. [30], where the values obtained after the optimization of the germination process reached 2900 µmol TE 100 g^−1^, and with those observed by Martín-Diana et al. [43], where the values obtained after optimizing the enzymatic hydrolysis process reached 7100 µmol TE 100 g^−1^.

**Figure 4:**
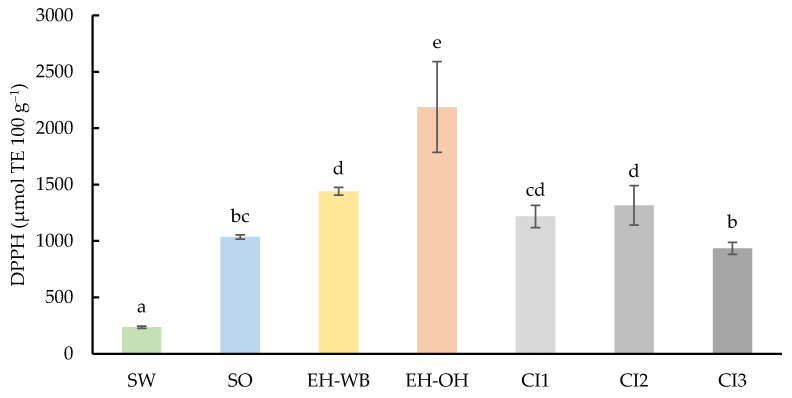
DPPH values of individual (SW, SO, EH-WB, and EH-OH) and combined ingredient (CI1, CI2, and CI3) samples. Results are expressed as µmol TE 100 g^−1^ of sample. Mean values are represented as bars, and standard deviations are represented as error bars. Different letters indicate significant differences from each other (one-way ANOVA, post hoc Duncan’s test, *p* ≤ 0.05). Abbreviations: sprouted wheat (SW), sprouted oat (SO), wheat bran hydrolysate (EH-WB), oat hull hydrolysate (EH-OH), combined ingredient 1 (CI1), combined ingredient 2 (CI2), and combined ingredient 3 (CI3).

Also, the activity against the DPPH radicals of the different ingredients and combinations was also evaluated (Figure 4), showing similar behavior to ORAC. The values obtained ranged from 235.54 to 2188.32 µmol TE 100 g^−1^, with significant differences (*p* < 0.05) observed among the different samples. The combined ingredients yielded intermediate values against the DPPH radicals, although with few differences between them. These values are in line with those previously reported by the authors [30,43], who obtained values higher than 350 µmol TE 100 g^−1^ for the sprouts and higher than 1300 µmol TE 100 g^−1^ for the hydrolysates. Although the DPPH radical test is less specific than the ABTS radical assay, it has interesting advantages such as its stability as an artificial radical and its usefulness in similarly neutralizing peroxyl radical reactions [72]. This property is useful to determine the antioxidant capacity of especially phenolic compounds present in the samples and to associate them with the prevention of oxidative stress reactions in which peroxyl radicals are implicated, such as lipid peroxidation reactions [73].

**Figure 5:**
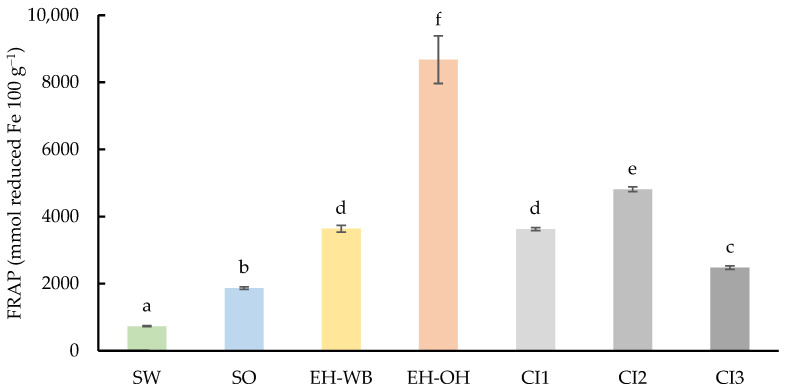
FRAP values of individual (SW, SO, EH-WB, and EH-OH) and combined ingredient (CI1, CI2, and CI3) samples. Results are expressed as mmol Fe^+2^ equivalents 100 g ^−1^ of sample. Mean values are represented as bars, and standard deviations are represented as error bars. Different letters indicate significant differences from each other (one-way ANOVA, post hoc Duncan’s test, *p* ≤ 0.05). Abbreviations: sprouted wheat (SW), sprouted oat (SO), wheat bran hydrolysate (EH-WB), oat hull hydrolysate (EH-OH), combined ingredient 1 (CI1), combined ingredient 2 (CI2), and combined ingredient 3 (CI3).

FRAP (ferric reducing antioxidant power) was evaluated since it is a widely used method to assess the antioxidant capacity of biological samples. It measures the ability of an antioxidant to reduce ferric ions (Fe^3+^) to ferrous ions (Fe^2+^), thus indicating its potential to neutralize free radicals and prevent oxidative damage in the body. The reducing power of the samples as evaluated using the FRAP assay is shown in Figure 5. Significant differences (*p* < 0.05) were observed among the results obtained in this assay, with values ranging from 734.02 to 8674.51 mmol reduced Fe 100 g^−1^.

Previous studies have shown values between 1500 and 1900 mmol reduced Fe 100 g^−1^ for FRAP in sprouts, while for hydrolysates, values have reached 3000 mmol reduced Fe 100 g^−1^ [20,74]. The reducing power of the ingredients showed a similar response to that observed with the ORAC and DPPH assays. CI2 was the combined ingredient with the highest reducing power.

A comprehensive evaluation, by integrating results from all the in vitro methods used (RACI), was used to obtain a more complete and accurate assessment of the antioxidant capacity and provide a robust framework for studying the interactions between different antioxidants and their combined effects. Table 2 shows the RACI value of single and combined ingredients. The highest RACI values for single ingredients corresponded to hydrolyzed oat bran, followed by hydrolyzed wheat bran, and the combined CI2 was significantly higher that CI1 and CI3. 

### 3.3. Total Starch Content (TSC) and Glycemic Index (GI)

**Figure 6:**
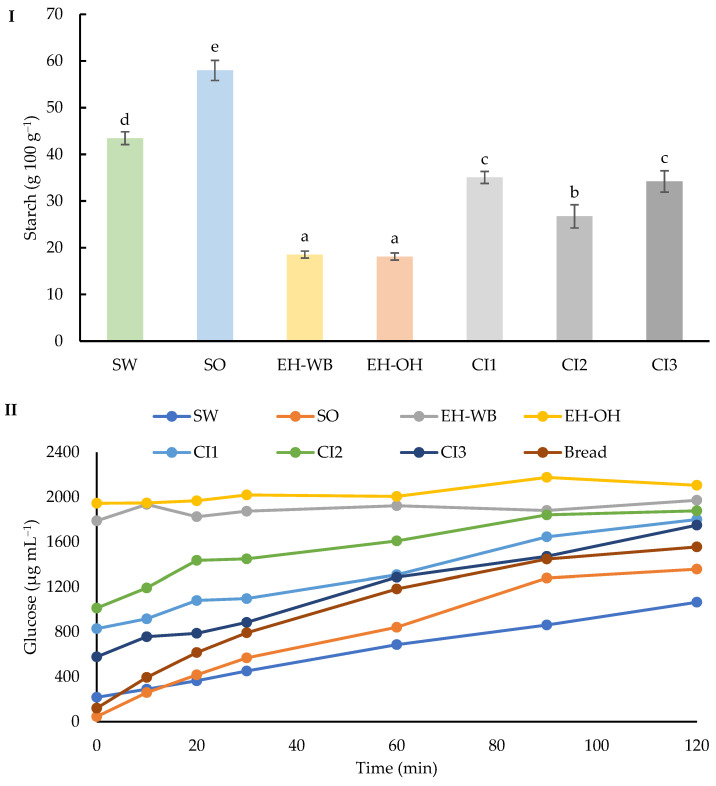
Starch (**I**) values and glucose kinetics consumption (**II**) for individual ingredient (SW, SO, EH-WB, and EH-OH) and combined ingredient (CI1, CI2, and CI3) samples. Results are expressed as g 100 g^−1^ of sample for starch and µg glucose mL^−1^ for glucose kinetics consumption. Bars represent means, and error bars represent standard deviations. Different letters indicate significant differences from each other (one-way ANOVA, post hoc Duncan’s test, *p* ≤ 0.05). Abbreviations: sprouted wheat (SW), sprouted oat (SO), wheat bran hydrolysate (EH-WB), oat hull hydrolysate (EH-OH), combined ingredient 1 (CI1), combined ingredient 2 (CI2), and combined ingredient 3 (CI3).

The TSC of the different samples is shown in Figure 6I. The values obtained ranged from 18.12 to 57.99 g 100 g^−1^, with significant (*p* < 0.05) differences among the samples. The combination of ingredients led to an intermediate starch content compared to the raw ingredients, with CI2 being the combined ingredient exhibiting the lowest starch content. The results obtained for the raw ingredients were similar to those previously reported for sprouts (ranging from 50 to 60 g 100 g^−1^) [20,75], while for hydrolysates, as a result of enzymatic action and degradation, the values were higher than those reported for wheat bran and oat hull [16,20]. Starch values are relevant for their association with the glycemic index of the final ingredient. 

The glycemic index (GI) of the individual and combined ingredients was estimated due to the importance of glycemic control of the ingredients and their potentially health-promoting properties (Table 3). The glucose kinetic curves are shown in Figure 6II. Although the combined ingredients initially exhibited a higher glucose concentration compared to white bread (control), their final glucose concentration was similar to that of the control. The glucose liberation of the combined ingredients was mitigated over time despite the high initial glucose content.

The IG values of the combined ingredients were higher than the individual ones, but lower than the expected values according to the ratio when the ingredients were formulated using a ratio 2:1 of hydrolyzed components (CI2) or double the amount of germinated components (CI3). The reduction in the IG was close to 20–25%. These results suggest the presence of phenolic and β-glucan compounds which are able to control the glucose response by different mechanisms [76]. The germinated ingredients showed lower IG than the hydrolyzed ones, as was expected, since although the germination promotes the hydrolysis of starch into small and simple sugars, the hydrolyzed components had a most significant effect of this effect. Probably, this is due to phenolic compounds such as ferulic acid or avenanthramides, which may have an inhibitory effect on α-amylase activity [77,78].

### 3.4. β-Glucan and Phytic Acid (PA)

**Figure 7:**
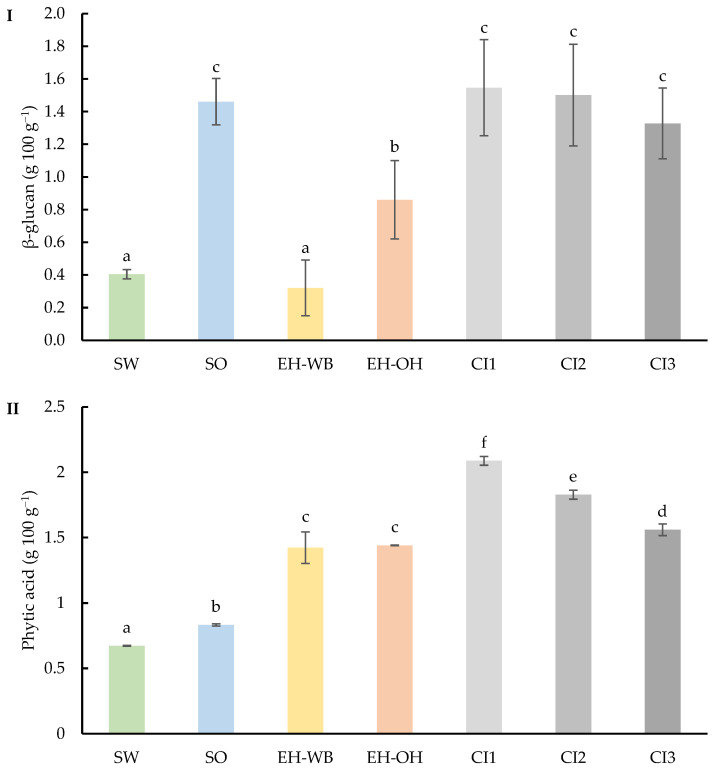
β-glucan (**I**) and phytic acid (**II**) values of different ingredient (SW, SO, EH-WB, and EH-OH) and combined ingredient (CI1, CI2, and CI3) samples. Results are expressed as g 100 g^−1^ of sample. Mean values are represented as bars, and standard deviations are represented as error bars. Different letters indicate significant differences from each other (one-way ANOVA, post hoc Duncan’s test, *p* ≤ 0.05). Abbreviations: sprouted wheat (SW), sprouted oat (SO), wheat bran hydrolysate (EH-WB), oat hull hydrolysate (EH-OH), combined ingredient 1, 2, and 3 (CI1, CI2, and CI3, respectively).

The β-glucan content was determined in both the individual ingredients and their combined forms due to its importance as a bioactive compound associated with various beneficial health effects [79]. The β-glucan content ranged from 0.32 to 1.55 g 100 g^−1^, with significant differences (*p* < 0.05) among the different samples (Figure 7I). The combined ingredients reached similar values to SO, with no significant differences observed between the three studied combinations. These findings are in line with other studies where germinated oat grains decreased in their β-glucan content, compared to ungerminated grains, with values close to 2 g 100 g^−1^ [80,81], while for hydrolyzed grains, this value reaches 1.5 g 100 g^−1^ [20].

The phytic acid (PA) content of the samples was also measured (Figure 7II), as it is considered to be one of the major anti-nutrients present in cereals. Significant differences (*p* < 0.05) were observed between the values obtained for PA, which ranged from 0.67 to 2.09 g 100 g^−1^. The combined ingredients showed a higher PA content, especially CI1, suggesting a cumulative effect of this anti-nutrient. The values obtained are similar to those previously found for sprouts [20] and lower than those found in bran [20,41]. However, the capacity of PA to link with mineral ions allows the inhibition of α-amylase activity, which is able to reduce the in vitro digestibility of starch and consequently the GI [82,83].

### 3.5. Anti-Inflammatory Activity

**Figure 8:**
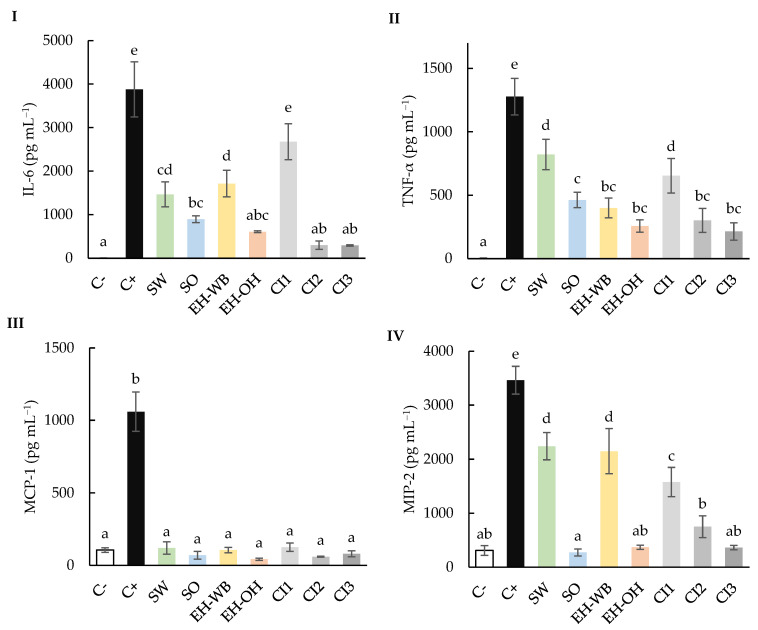
Evaluation of (**I**) IL-6, (**II**) TNF-α, (**III**) MCP-1, and (**IV**) MIP-2 production in RAW264.7 macrophages treated with C- (complete DMEM), C+ (1 µg mL^−1^ of LPS), or LPS-stimulated macrophages and treated with sprouted wheat (SW), sprouted oat (SO), wheat bran hydrolysate (EH-WB), oat hull hydrolysate (EH-OH), and combined ingredient 1, 2, and 3 (CI1, CI2 and CI3, respectively) at 0.5 mg mL^−1^ for 24 h. Bars represent means, and error bars represent standard deviations (n = 4). Different letters indicate significant differences from each other (one-way ANOVA, post hoc Duncan’s test, *p* ≤ 0.05). Abbreviations: interleukin (IL), tumor necrosis factor-alpha (TNF-α), monocyte chemoattractant protein-1 (MCP-1), and macrophage inflammatory protein-2 (MIP-2).

This study evaluated the individual and combined anti-inflammatory properties (in inhibition of IL-6, TNF-α, MCP-1, and MIP-2 production) of sprouted and hydrolyzed oat and wheat ingredients using LPS-stimulated RAW264.7 macrophages (Figure 8I–IV). All ingredients significantly (*p* ≤ 0.05) reduced the production of the selected pro-inflammatory cytokines compared to the control. Only in the case of IL-6, CI1 achieved a non-significant (*p* > 0.05) reduction. These results are consistent with previous studies demonstrating the anti-inflammatory properties of both sprouted and hydrolyzed oat [20] and wheat [74] ingredients, similarly evaluated using LPS-stimulated RAW264.7 macrophages as an in vitro model of inflammation.

The combined ingredients CI2 and CI3, along with oat hull hydrolysate (~84%), exhibited the most potent IL-6 inhibition (~92%) (Figure 8I). Similar trends were observed for TNF-α inhibition; the hydrolysates (EH-WB and EH-OH) and the combined ingredients 2 and 3 exhibited the lowest cytokine values. CI3 demonstrated the strongest inhibition, with approximately an 83% reduction in cytokine expression compared to the control (Figure 8II). The strongest anti-inflammatory effect obtained from the ingredients was observed in MCP-1 expression, where all ingredients significantly reduced MCP-1 levels to those of the negative control, with EH-OH showing a remarkable inhibition of 96% (Figure 8III). Finally, the lowest production of the MIP-2 cytokine was observed when RAW264.7 macrophages were treated with sprouted oat (92% of inhibition), followed by CI3 and EH-OH with ~90% and ~89% of inhibition, respectively (Figure 8IV).

Interestingly, when separately comparing sprouted (SW vs SO) or hydrolysates (EH-WB vs EH-OH), oat ingredients generally exhibited higher anti-inflammatory effects compared to wheat. This may be attributed to their higher β-glucan content (Figure 7I), a known anti-inflammatory compound [84,85]. Although EH-OH had a lower β-glucan content than SO, it surpassed wheat-based ingredients (SW and EH-WB), and EH-OH also showed the highest TP content and antioxidant properties. On the other hand, EH-WB had double the phytic acid content of the sprouted ingredients (SW and SO), as did EH-OH. This result, along with the reduced β-glucan content, could explain the lack of correlation between EH-WB’s antioxidant properties (relatively high) and anti-inflammatory results (relatively weak) [85]. This effect may explain the observed effect in combined ingredients, as those with higher proportions of hydrolysates (CI2) or sprouts (CI3) exhibited higher cytokine inhibition than CI1. In addition, there was a higher (although not significant) anti-inflammatory effect in CI3. In the case of MIP-2 expression, this resulted significantly as low as the negative (no inflammation) control MIP-2 levels.

Additionally, the lack of significant differences (*p* > 0.05) in β-glucan content between the combined ingredients (Figure 7I), along with the higher content of TPs in CI1 and CI2 compared to CI3, suggests the presence of additional bioactive anti-inflammatory compounds. These might include bioactive peptides, xylooligosaccharides, found in wheat [74,86], and avenanthramides, phenolic compounds present in oats [20]. Previous studies have shown that avenanthramide supplementation reduces the expression of pro-inflammatory genes (TNF-α, IL-6, and NF-κB) in mice fed with a high-fat diet [87]. In addition, bioactive peptides derived from wheat protein may exert anti-inflammatory properties by modulating immune cell signaling or scavenging reactive oxygen species (ROS) which contribute to inflammation.

## 4. Conclusions

Most nutraceutical studies are focused on the analysis of individual ingredients; however, there is an important research gap regarding the effects of combined ingredients. It is important to consider the combined effects of ingredients in products, as their interactions can have a significant impact on their overall efficacy. The effects of combined ingredients could provide valuable insights into optimizing product formulations and improving their overall performance. In conclusion, investigating the effects of combined ingredients is an important area of study that could help to advance our understanding of how different components interact and work together in different products. By exploring the synergies between ingredients, researchers can uncover new opportunities to create more innovative and impactful products for consumers.

The results obtained suggest that the combination of individual ingredients developed through both proposed biotechnological strategies allows obtaining a nutraceutical ingredient with a high content of polyphenols, which may be responsible for most of the antioxidant capacity observed. However, protein mobilization during germination and the release of peptides may contribute to the increase in antioxidant properties, as evaluated by the ABTS radical assay of the germinated ingredients SW and SO. The antioxidant properties observed in the combined ingredients were proportional to the ratio of individual ingredients, with no interaction or synergy observed between individual ingredients.

However, the anti-inflammatory results showed an interesting behavior, with a potentially synergetic effect of the individual ingredients. This effect was observed in CI2 and CI3, resulting in a higher ability to inhibit IL-6 and TNF-α than was expected from the anti-inflammatory values of their individual ingredients. As with the antioxidant properties, the oat-based ingredients contributed significantly higher to the anti-inflammatory properties of the ingredients, probably related to the β-glucans and avenanthramides present in oats, which have been described for their ability to inhibit TNF-α by modulating the activity of immune cells, such as macrophages, and their capacity to stimulate the production of anti-inflammatory cytokines, contributing to the regulation of the immune response and reduction in inflammation [88,89,90,91].

The mechanisms of action of antioxidants and β-glucans present in the nutraceutical ingredients from cereals involve several pathways, including the inhibition of oxidative enzymes and the activation of antioxidant enzymes. These antioxidants also have the ability to chelate metal ions that can contribute to oxidative stress in the body. By reducing oxidative stress and inflammation, bran antioxidants may help protect against chronic diseases such as cancer, cardiovascular disease, and neurodegenerative disorders.

In summary, bran antioxidants work by scavenging free radicals, reducing oxidative stress, and regulating inflammation in the body. By increasing antioxidant activity and reducing inflammatory properties, the bioactive compounds found in bran can help to promote overall health and protect against chronic diseases.

In order to evaluate the bioaccessibility of these ingredients, further studies including simulated digestion protocols would be required.

## Figures and Tables

**Table 1:** Proportion by weight of each ingredient in the processed combined ingredients.

	EH-WB	SW	EH-OH	SO
**Combined Ingredient 1 (CI1)**	1	1	1	1
**Combined Ingredient 2 (CI2)**	2	1	2	1
**Combined Ingredient 3 (CI3)**	1	2	1	2

**Abbreviations:** wheat bran hydrolysate (EH-WB), sprouted wheat (SW), oat hull hydrolysate (EH-OH), and sprouted oat (SO).

**Table 2:** RACI values for individual ingredient (SW, SO, EH-WB, and EH-OH) and combined ingredient (CI1, CI2, and CI3) samples.

	RACI
**SW**	−1.15 ± 0.05 ^a^
**SO**	−0.85 ± 0.07 ^b^
**EH-WB**	0.28 ± 0.06 ^e^
**EH-OH**	1.89 ± 0.11 ^f^
**CI1**	−0.11 ± 0.06 ^d^
**CI2**	0.28 ± 0.22 ^e^
**CI3**	−0.34 ± 0.02 ^c^

Abbreviations: wheat bran hydrolysate (EH-WB), sprouted wheat (SW), oat hull hydrolysate (EH-OH), and sprouted oat (SO). Small letters indicate significant differences between RACI values using one-way ANOVA.

**Table 3:** Estimated glycemic index (GI) values for individual ingredient (SW, SO, EH-WB, and EH-OH) and combined ingredient (CI1, CI2, and CI3) samples.

	GI
**SW**	32.11 ± 0.14 ^a^
**SO**	39.59 ± 6.63 ^b^
**EH-WB**	96.07 ± 3.04 ^f^
**EH-OH**	100.00 ± 8.36 ^f^
**CI1**	67.33 ± 2.53 ^d^
**CI2**	79.08 ± 3.00 ^e^
**CI3**	60.94 ± 0.01 ^c^

Results were expressed as mean ± standard deviation. Different letters indicate significant differences from each other (one-way ANOVA, post hoc Duncan’s test, *p* ≤ 0.05). Abbreviations: sprouted wheat (SW), sprouted oat (SO), wheat bran hydrolysate (EH-WB), oat hull hydrolysate (EH-OH), combined ingredient 1 (CI1), combined ingredient 2 (CI2), and combined ingredient 3 (CI3).

## Data Availability

The original contributions presented in the study are included in the article, further inquiries can be directed to the corresponding author.
